# Magnetic fingerprint of individual Fe_4_ molecular magnets under compression by a scanning tunnelling microscope

**DOI:** 10.1038/ncomms9216

**Published:** 2015-09-11

**Authors:** Jacob A.J. Burgess, Luigi Malavolti, Valeria Lanzilotto, Matteo Mannini, Shichao Yan, Silviya Ninova, Federico Totti, Steffen Rolf-Pissarczyk, Andrea Cornia, Roberta Sessoli, Sebastian Loth

**Affiliations:** 1 Max Planck Institute for the Structure and Dynamics of Matter, 22761 Hamburg, Germany; 2 Max Planck Institute for Solid State Research, 70569 Stuttgart, Germany; 3 Department of Chemistry ‘Ugo Schiff', University of Florence & INSTM RU of Florence, 50019 Sesto Fiorentino, Italy; 4 Department of Chemical and Geological Sciences, University of Modena and Reggio Emilia & INSTM RU of Modena and Reggio Emilia, 41125 Modena, Italy

## Abstract

Single-molecule magnets (SMMs) present a promising avenue to develop spintronic technologies. Addressing individual molecules with electrical leads in SMM-based spintronic devices remains a ubiquitous challenge: interactions with metallic electrodes can drastically modify the SMM's properties by charge transfer or through changes in the molecular structure. Here, we probe electrical transport through individual Fe_4_ SMMs using a scanning tunnelling microscope at 0.5 K. Correlation of topographic and spectroscopic information permits identification of the spin excitation fingerprint of intact Fe_4_ molecules. Building from this, we find that the exchange coupling strength within the molecule's magnetic core is significantly enhanced. First-principles calculations support the conclusion that this is the result of confinement of the molecule in the two-contact junction formed by the microscope tip and the sample surface.

## Abstract (web summary)

The incorporation of single-molecule magnets into spintronic devices is often hindered by electronic or structural modifications. Here, the authors demonstrate how confinement of Fe_4_ molecules in junctions between a Cu_2_N substrate and a scanning microscope tip enhances intra-molecular exchange interaction.

## Graphical abstract

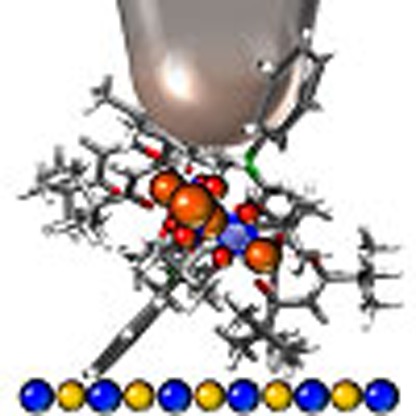

Molecular spintronics harnesses magnetic properties of molecules to achieve enhanced functionality in electronic circuits^1^. The use of single-molecule magnets (SMMs) with long spin-relaxation times may even enable spin-based quantum computing^2^,^3^. Practical incorporation of an SMM into a two-contact device demands strong coupling to the spin subsystem without disrupting the magnetic properties with unwanted electronic or structural modifications^4^,^5^,^6^,^7^,^8^. Metal-molecule-metal junctions constructed by electro-migration have demonstrated the coupling between electric current and molecular magnetic moments, as well as the persistence of magnetic anisotropy within the device junction for [Mn_12_O_12_(O_2_CR)_16_(H_2_O)_4_] (Mn_12_), [Fe_4_(L′)_2_(dpm)_6_] and TbPc_2_ SMMs^9^,^10^,^11^,^12^. Indirect electronic contact to TbPc_2_ using carbon-nanotube and graphene-based devices showed signs of slow magnetization dynamics^13^,^14^. However, on metallic surfaces the magnetic bistability of TbPc_2_ is quenched^4^,^5^,^6^ and recovers only for significant separation of molecule and surface^7^. Mn_12_ proved to be exceedingly fragile^8^ unless the molecules are isolated by a protective layer^15^. In contrast, our focus here is directed to the four exchange coupled Fe atoms of the tetrairon(III) (Fe_4_) system which are encased in a robust, rigid, three-dimensional (3D) organic ligand shell. Fe_4_ allows significant flexibility in customization of ligands^16^ and stable magnetism has been detected in ensembles of both chemically grafted^17^,^18^ and sublimated^19^,^20^ Fe_4_ derivatives on metallic surfaces.

Here, we use a low-temperature scanning tunnelling microscope (STM) to study the magnetic properties of Fe_4_ molecules sublimated onto the surface of a Cu_2_N/Cu(100) substrate. The STM tip is used to address individual Fe_4_ molecules and probe excitations of the Fe_4_ SMM's electron spin by inelastic electron tunnelling spectroscopy (IETS)^21^. Hindering this is the 3D nature of the molecule and its ligand shell. In particular, tip interaction during spectroscopic measurements is extremely strong, commonly leading to molecular fragmentation. We overcome this problem by implementing analysis that correlates independent metrics (topography and IETS spectrum) to categorize the magnetic fragments on the surface and identify intact molecules. Analysis of the spectrum identified for intact Fe_4_ molecules shows that the exchange energy within the magnetic core of the molecule is boosted by a significant margin. From first-principles calculations, we find that the enhanced exchange interaction between the Fe ions can be explained by a small compression of the magnetic core. We attribute this compression to the confinement of the molecule in the two-contact junction formed by the STM tip and the substrate surface.

## Results

### Fe_4_ evaporated on Cu_2_N

**Figure 1:**
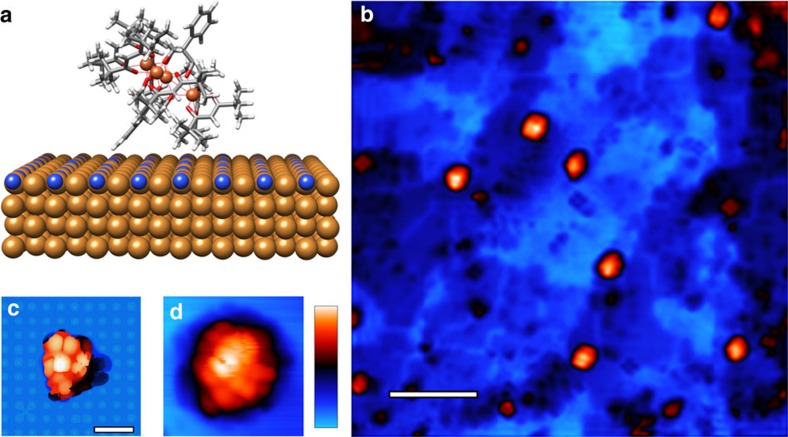
Fe_4_ molecule adsorbed on the Cu_2_N surface. (**a**) [Fe_4_(L)_2_(dpm)_6_] resting on the Cu_2_N surface. Adsorption geometry and molecular structure are computed by density function theory (DFT). The molecule's axis defined by the tripodal ligands is at a 33° angle from the surface normal. Atoms are Fe (orange), O (red), C (grey), H (white), Cu (brown) and N (blue). (**b**) Overview scanning tunnelling microscope (STM) image of the Cu_2_N surface after deposition of molecules (scale bar, 8 nm). A number of Fe_4_ molecules are visible as the tallest objects in orange. This image was filtered to remove noise using WSxM software^23^. (**c**) Calculated top view image of relaxed Fe_4_ on Cu_2_N showing the spatial distribution of the density of states integrated between 0 and +3 eV in energy. (**d**) STM image of an Fe_4_ molecule. It appears as a spheroid of ∼2 nm diameter with a multi-lobed substructure consistent with the calculated image in (**c**). The colour scale indicates the topographical height ranging between 0 and 1 nm in (**b**,**d**), which were acquired at a tunnel current set-point of 3 pA and bias voltage of 2.3 V. The 1-nm lateral scale bar inset in (**c**) also applies to (**d**).

The Fe_4_ derivative used in this work ([Fe_4_(L)_2_(dpm)_6_], where H_3_L is the tripodal ligand Ph-C(CH_2_OH)_3_ and Hdpm is dipivaloylmethane; Fig. 1a) permits thermal sublimation of isolated molecules onto a semi-insulating copper nitride (Cu_2_N) surface on Cu (100) that has been pre-cooled (see Methods for details)^19^,^20^.

After deposition onto the Cu_2_N surface, and immediate cooling below 1 K, constant-current topographs reveal molecular objects with a wide variety of morphologies (Fig. 1b). Density functional theory (DFT) computations (see Methods for details) of Fe_4_ relaxed on a Cu_2_N slab indicate that molecules adsorb with the axis of approximate 3-fold rotational symmetry (pseudo-*C*_3_ axis) canted at a 33° angle from the surface normal, and with a total height of 1.7 nm (Fig. 1a,c). The tallest objects on the surface appear as spheroids of width 2 nm and height between 700 and 800 pm in STM scans (Fig. 1d). Unambiguously identifying these objects as intact Fe_4_ SMMs is not possible by topographic measurements alone because the fine, multi-lobed structure is strongly tip-dependent.

### Spectroscopic measurements

**Figure 2:**
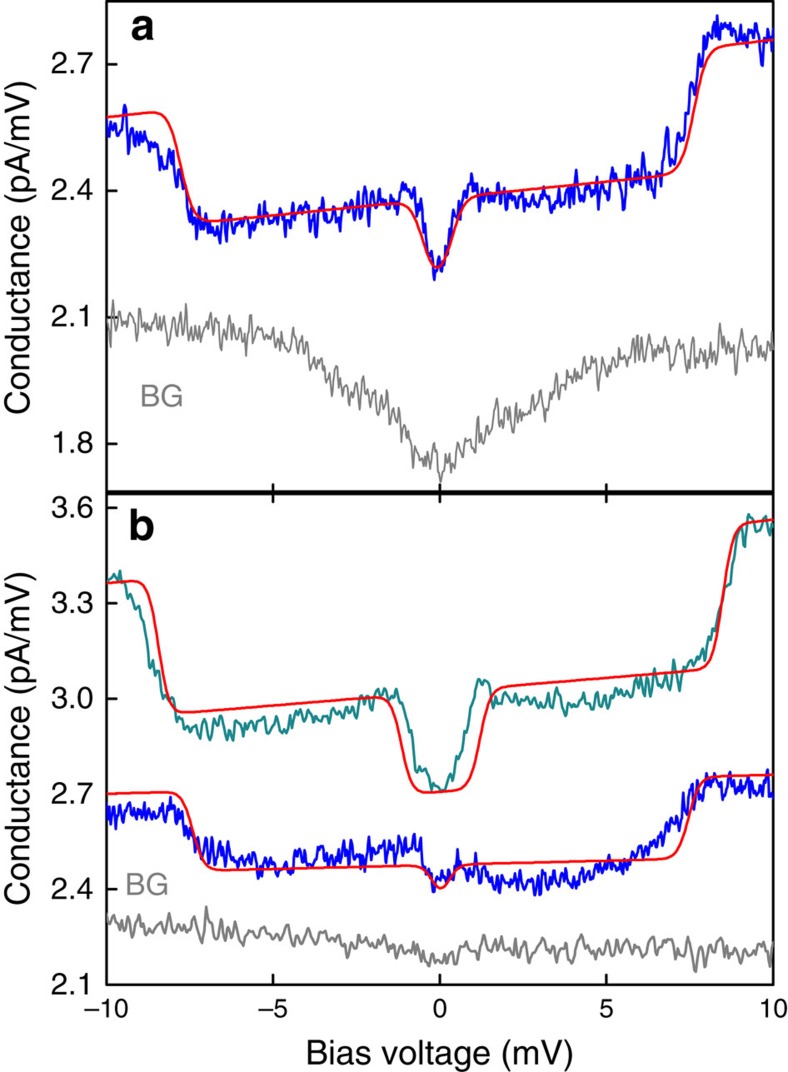
Conductance spectra of individual molecules. (**a**) d*I*/d*V*(*V*) spectrum recorded on a molecule with 700 pm topographic height at 0 T magnetic field (blue line, initial current *I*_*o*_=75 pA at *V*_*o*_=10 mV). A background (BG) spectrum recorded with the same tip on bare Cu_2_N is shown in grey. (**b**) Spectra acquired on a single molecule (800 pm height) without magnetic field (blue line, *I*_*o*_=25 pA, *V*_*o*_=10 mV) and under a 9 T out-of-plane field (green line, *I*_*o*_=50 pA, *V*_*o*_=15 mV), background spectrum (BG, grey line). When a magnetic field is applied, the low-energy excitation widens from 0.2±0.2 to 0.8±0.1 mV and the high-energy excitation widens from 7.5±0.1 to 8.5±0.1 mV. Background spectra in (**a**) and (**b**) are offset for clarity. Red lines in (**a**) and (**b**) indicate spectra computed using the spin Hamiltonian model. The fits yield exchange coupling, *J*=2.93 meV (23.6 cm^−1^), and magnetic anisotropy, *D*=−52 μeV (−0.42 cm^−1^) for the molecule in (**a**) and *J*=2.89 meV (23.3 cm^−1^), *D*=−26 μeV (−0.21 cm^−1^) with a *g* factor of 2 for the molecule in (**b**).

To corroborate the coarse topographic match we record inelastic electron tunnelling spectra with the tip brought into contact with the molecules. The differential conductance, d*I*/d*V*, is measured as a function of bias voltage, *V*, starting at reduced bias voltage (10 mV) and increased tunnel current (5–100 pA; see Methods). Over a molecule at typical scanning conditions (2 V, 3 pA), we estimate the tip-Cu_2_N gap to be 1.5±0.1 nm (see Supplementary Fig. 1 and Supplementary Note 1); this matches well with the expected molecule height. Under scanning conditions transient physical tip-molecule interactions occur, confirming that the tip comes into contact with the top of the molecule. Transitioning to spectroscopy conditions moves the tip towards the molecule by 700–800 pm (see Supplementary Fig. 1). Hence, current passes directly through the molecule, as it is sandwiched into a two-contact device formed by the STM junction. Figure 2a shows a representative spectrum acquired on one of the molecules so trapped. It features clear steps in d*I*/d*V*(*V*) at ±0.5 mV and at ±7.5 mV that stem from excitations of the molecule's electron spin. We verify the magnetic nature of these excitations by acquiring data on individual molecules at both zero field and under an out-of-plane 9 T field, Fig. 2b. Both excitations shift in energy with magnetic field, qualitatively consistent with Zeeman energies expected for electron spins in a 9 T magnetic field.

**Figure 3:**
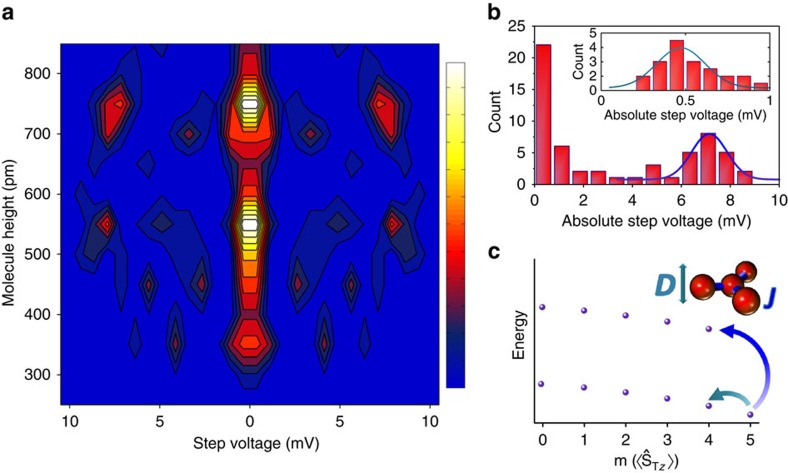
Spin excitation fingerprint of Fe_4_ on Cu_2_N. (**a**) Two-dimensional histogram for an ensemble of molecules (>60) correlating zero-field spin excitation energies of a molecule with its topographic height. The histogram counts the number of inelastic tunnelling steps observed in d*I*/d*V*(*V*) binned by step voltage and topographic height of the molecule measured after completion of each spectrum. For molecules over 700 pm in height, a dominant spectrum can be identified. For shorter objects large variation indicates significant changes in magnetic structure and possible fragmentation. Bin sizes are 0.75 mV and 50 pm. The colour scale indicates bin count between 1 and 11. Since spin excitations are symmetric with respect to 0 V, the absolute value of the steps is used, and the symmetrized plot is shown. (**b**) Histogram of all steps measured on molecules >700 pm in height. Focusing on those molecules reveals the characteristic spectrum for intact Fe_4_ molecules, which features two spin excitations: a low-energy excitation at 0.47 mV and a high-energy excitation at 7.2 mV. Both have broad distributions with standard deviations of 0.14 and 0.7 mV, respectively reflecting variations in spin excitation energies for different molecules. (**c**) Spin state distribution for Fe_4_ single-molecule magnets. States are calculated using a model of the magnetic core (inset) incorporating antiferromagnetic exchange coupling of the three outer Fe ions to the central ion (orange balls) with strength *J* (blue bonds) and easy-axis magnetic anisotropy with strength *D* applied to the whole molecule (teal arrow). The characteristic excitations found in (**b**) are consistent with the two lowest energy transitions excitable by inelastic electron tunnelling (curved teal and blue arrows) with *J* between 2.5 meV (20 cm^−1^) and 3.1 meV (25 cm^−1^), and *D* between −34 μeV (−0.28 cm^−1^) and −86 μeV (−0.70 cm^−1^).

Different molecules, however, exhibit a large variation of the spin excitation energies. It is not, *a priori*, clear if these variations might stem from measurements on partially fragmented molecules or interaction with the electrodes. This uncertainty can be overcome by correlating topographic height with observed spin excitations over a statistically significant population of molecules (>60). Figure 3a shows a 2D histogram of the spin excitation steps detected at zero magnetic field, binned by excitation voltage and topographical height of each molecule, measured following the spectroscopic measurement. Since, spin excitations are symmetric in energy, we plot symmetrized data (for comparison see nonsymmetrized data in Supplementary Fig. 2). Clear groupings of excitations appear correlated with height. In particular a distinct set of peaks is visible for molecules taller than 700 pm (Fig. 3b), corresponding to two spin excitations with characteristic energy Δ*E*_1_=0.47 meV and Δ*E*_2_=7.2 meV, and distribution standard deviations of 0.14 and 0.7 meV, respectively. The breadths of the observed distributions are larger than the experimental error on excitation energies (0.1 meV), therefore they represent variation in the expression of the molecule in the junction. This emergent spectrum is linked to molecules that feature a coarse topographical match with the computed DFT structure, thus revealing the spin excitation fingerprint of Fe_4_ on Cu_2_N.

Among shorter molecules measured in Fig. 3a, the peaks are more scattered, supporting the hypothesis that these are disrupted or fragmented molecules. The exact chemical configuration of the fragments remains unknown but the most commonly observed spectra qualitatively match those expected for clusters comprising two and three Fe atoms with coupling parameters similar to intact molecules (see Supplementary Fig. 3 and Supplementary Note 2). The potentially insidious role magnetic fragments may play in the interpretation of experiments on bulky, fragile molecules can thus be mitigated by identifying intact molecules via correlation of independent metrics, such as spectral information and topographic height.

### Modelling of spin excitations

Magnetic properties of Fe_4_ are quantitatively investigated by fitting the spin excitation energies extracted from Fig. 3b to an effective spin Hamiltonian that incorporates the dominant exchange coupling between the central Fe ion and the side ions and net second-order uniaxial anisotropy (equation 1). Less influential contributions from next-nearest neighbour exchange, rhombic anisotropy and higher-order anisotropies are neglected. The energy eigenstates of the simplified Hamiltonian deviate by less than the measurement accuracy from a more complex spin Hamiltonian reported previously^16^ (see Supplementary Note 3 and Supplementary Fig. 4).


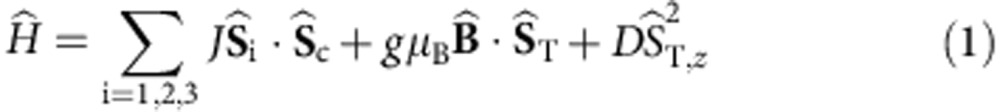



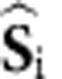
 and 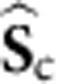 denote the spin vector operators for the three side ions and for the central ion respectively, where each ion has a spin 5/2. The total spin operator of the molecule, 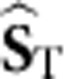, is given by 
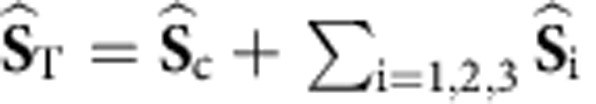
 and 
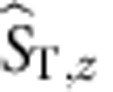
 is its component along the easy magnetic axis (*z*). Antiferromagnetic Heisenberg exchange coupling, *J*>0 (Fig. 3c inset), is mediated by the oxygen bridges that connect the central and side ions and leads to a ferrimagnetic configuration and a *S*_T_=5 ground state. The next higher energy multiplet features *S*_T_=4; other multiplets lie at still higher energies. Uniaxial anisotropy, *D*, parallel to the tripodal ligands, splits spin states in each multiplet into a parabolic distribution typical of easy-axis (*D*<0) molecular magnets (Fig. 3c). A simplifying assumption that each multiplet shares the same *D* value is made (see Supplementary Note 3). Zeeman energy is included for spectra recorded at 9 T field; *g* is the Landé *g* factor and *μ*_B_ is the Bohr magneton. The out-of-plane magnetic field, **B**, is tilted by 33° from *z* to account for the orientation of the molecule on Cu_2_N.

Spin excitations with tunnelling electrons obey the selection rule *Δm*={+1, 0, −1} (*ℏΔm* is the change in the expectation value, *ℏm*, of the 
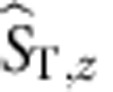
 operator)^22^. At 0.5 K, only the ground doublet with *m*=±5 is occupied. Therefore, the two characteristic excitations found for Fe_4_ can be linked to specific spin transitions: *ΔE*_1_ is a low-energy transition within the *S*_T_=5 spin multiplet from *m*=±5 to ±4, and Δ*E*_2_ is a transition to the lowest-lying states of the *S*_T_=4 multiplet. Fitting Δ*E*_1_ and Δ*E*_2_ to the above spin Hamiltonian yields a uniaxial anisotropy energy of *D*=−60±26 μeV (−0.48±0.21 cm^−1^) and exchange coupling energy of *J*=2.8±0.3 meV (23±2 cm^−1^). We note that the standard deviations given here reflect the variation in *D* and *J* among the ensemble of molecules.

## Discussion

The *J* value observed for molecules sandwiched in the two-contact junction of the STM is significantly higher than for bulk samples of Fe_4_ molecules, where *J*=1.92 meV (15.5 cm^−1^)^16^ for the spin Hamiltonian in equation 1 (see Supplementary Note 3). A scenario with lower *J* leading to the observed steps in d*I/*d*V* would require *ΔE*_2_ to be an excitation into higher-lying multiplets such as *S*_T_=6 but this can be ruled out by the observed magnetic-field-dependent energy shift of *ΔE*_2_ (see Supplementary Note 4). Similarly, a change in the magnetic properties of the molecule due to a change in the molecule's redox state as predicted for other types of molecules^24^ is unlikely. Changing the redox state would require accessing either the highest occupied or lowest unoccupied molecular orbitals of the Fe_4_ ligand shell. These are separated from the Fermi energy by several electronvolts and are not accessible during the IETS measurements performed here. Consequently, there must be some other mechanism that enhances exchange within the molecule.

We explore the influence of the Cu_2_N surface on the magnetic properties of the molecule via DFT calculations. Starting with the relaxed geometry computed for Fe_4_ on Cu_2_N, the magnetic properties may be computed using the broken symmetry approach^25^ (see Methods for details on DFT calculations). The structural relaxation of the molecule is found to reduce the exchange to 1.50 meV (12.1 cm^−1^), ∼20% below the experimental value for bulk molecular crystals. Inclusion of the electronic effects of the substrate further reduces the exchange to 1.17 meV (9.4 cm^−1^). The most important information gained here is that the structural changes induced by the surface and the interaction of the substrate electron bath both feature the same qualitative trend. Hence, the influence of the substrate is not responsible for the boosted exchange.

**Figure 4:**
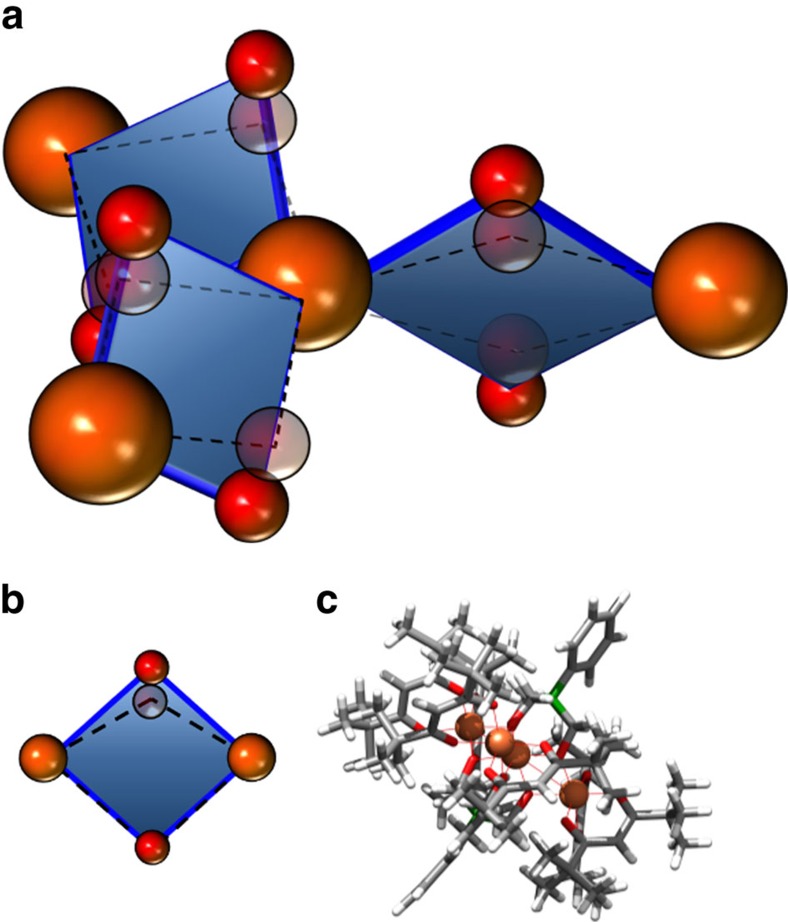
Structural distortions of Fe_4_ in the STM tunnel junction. The strong contact made by the tip induces distortions as the molecule is compressed in the STM junction and enhances super-exchange. (**a**) Schematic of the magnetic core of the Fe_4_ molecule. Key to the super-exchange coupling within the magnetic core are the Fe-O-Fe bond angles. The blue diamonds represent the planes of these bonds; oxygen atoms are red and iron atoms are orange. Compression of the molecule by the STM tip displaces the O atoms relative to the Fe atoms causing a tilt of the Fe_2_O_2_ bond planes and a change in the Fe-O-Fe bond angle. The distorted configuration is shown superimposed, semi-transparent with dashed lines. (**b**) Schematic depicting the small distortion applied for DFT calculation of exchange over a Fe_2_O_2_ unit. (**c**) Model of the compressed molecule used in DFT calculations. Atoms are Fe (orange), O (red), C (grey) and H (white). To simulate the compression inside the STM junction, the upper tripodal ligand (highlighted in green) is shifted downwards parallel to the molecule axis by 10 pm, equivalent to a 2% reduction in breadth of the magnetic core. This structural shift nearly doubles the computed exchange coupling.

We reason that the increase in *J* must be induced by the confinement of the molecule in the narrow STM junction. Hydrostatic compression has been found to alter the inter-molecular exchange in bulk crystals of organic ferromagnets^26^. In Fe_4_, exchange coupling between the central and side ions is predominantly mediated by the oxygen atoms of the tripodal ligands. A distortion of these ligands relative to the plane of the Fe ions provides a direct path to change the Fe-O-Fe angles^16^ and, consequently, the exchange coupling strength, *J*. We therefore consider the effect of distorting this ligand during compression of the molecule. We incorporate a rudimentary representation of this distortion into the DFT framework. Starting with the structure of the molecule, as relaxed on Cu_2_N, the upper tripodal ligand is shifted downwards, parallel to the axis of the molecule by 10 pm (Fig. 4). This is equivalent to compressing the molecular core by 2% of its starting breadth. Re-evaluating the exchange coupling and comparing to the value computed for the undistorted molecule isolated from the surface, we find that this minimal structural change increases *J* by a factor of 1.9, to a value of 2.82 meV (22.7 cm^−1^). The magnitude of the increase is sufficient to explain the boosted exchange interaction found for Fe_4_ in the STM junction.

In the experiment, the complexity of the situation is considerably greater. Along with other environmental effects there must be more complex distortions occurring. This is corroborated by the large variation of *D* and *J* observed for the ensemble of molecules (Fig. 3b). The 1σ standard deviation of Δ*E*_2_ relates to a variation in *J* of 10% (±0.3 meV). We therefore consider a range of other possible distortions (see Supplementary Fig. 5 and Supplementary Note 5) and find that rotating the tripodal ligand relative to the Fe ions and tilting of the protruding phenyl rings also affects *J*, albeit significantly less than the simple 2% compression (see Supplementary Tables 1 and 2). Other random contributions to the broad distribution of excitation energies are possible. No selection was made for differing positions on the Cu_2_N surface and it was not possible to inspect the surface beneath the molecules for the presence of defects, such as nitrogen vacancies. Consequently, a portion of the random variation may arise from local differences in substrate coupling, although this will be mitigated by the molecule's protective ligand shell. We additionally expect that the broad distribution found for Δ*E*_1_ reflects the influence of structural distortions on the molecular anisotropy. However, in contrast to the effect on exchange energy, no systematic shift was found experimentally.

Despite the broad distribution of parameters, individual Fe_4_ SMMs retain their qualitative magnetic character when incorporated into a prototypical two-contact device formed by an STM tunnel junction. Ascertaining this is made possible by performing an exhaustive survey of molecular objects. Correlation of spin excitation spectra with topographic height is a critical step in the study of bulky, polynuclear SMMs. By using this approach, we find that Fe_4_ molecules in the STM junction feature increased exchange coupling strength that can be accounted for by tip-induced structural distortions. While demonstrating that Fe_4_ may be incorporated into a prototypical device, this work also addresses key challenges in combining electronic transport devices with SMMs. Not only can the molecule's bulky ligand shell aid in the preservation the molecule's magnetic properties, but it can also participate in strong mechanical interactions with the electrodes. Confronting and overcoming these challenges facilitate the rational design of SMMs and creates the opportunity for identification of novel effects, such as the possibility of tuning intra-molecular properties by mechanical motion.

## Methods

### Experiment

Observations were made with an ultrahigh-vacuum low-temperature STM (Unisoku USM1300) equipped with a ^3^He cryostat operated at 0.5 K, and with a magnetic field of up to 9 T applied perpendicular to the sample surface. The copper crystal was cleaned using cycles of Ar sputtering (1 kV, pressure=5 × 10^−6^ mbar) and annealing (*T*=870 K) using an electron-beam heater built into the sample holder. The final annealing cycle used a lower temperature of 770 K. The copper nitride monolayer was formed by three minutes of nitrogen sputtering (1 kV, pressure=5 × 10^−6^ mbar) followed by annealing for 8 min at 640 K. Platinum iridium (90:10) tips were used to perform all STM measurements. Tips were prepared by 5 min of Ar sputter cleaning (1 kV, pressure=5 × 10^−6^ mbar) followed by heating using 10 s pulses of electron beam bombardment and a gentle dipping into the Cu crystal.

Fe_4_ molecules, synthesized and isolated in crystalline form^16^, were mechanically ground into a powder and sublimated from a home-built quartz Knudsen cell maintained at a constant temperature of 483 K. Deposition times ranged between 10 and 30 s. The Cu_2_N/Cu substrate was pre-cooled to 4 K in the STM cryostat, then moved rapidly to the deposition chamber and back to the cryostat; during this procedure the sample temperature reached an estimated maximum of 100 K.

### Acquisition of spin excitation spectra

Inelastic electron tunnelling spectra were acquired by positioning the tip over candidate molecules at typical scanning conditions (3 pA, 2.0–2.5 V). Subsequently, the bias voltage was reduced slowly to 10–15 mV, then the tunnel current set-point was increased to 5–100 pA. Following this procedure, the feedback loop was disengaged, and the sample bias voltage was swept. A small modulation voltage (100–300 μV) at 691 Hz was added to permit direct lock-in detection of differential conductance, d*I*/d*V*(*V*).

### Calculated spin excitation spectra

The conductance spectra were calculated from the eigenstates and eigenenergies of the effective spin Hamiltonian (equation 1) using a perturbative treatment of inelastic electron-spin scattering 27. In this approach, a spin excitation between state |2〉 and |1〉 with Δ*E* energy appears as steps in d*I*/d*V*(*V*) at +Δ*E*/e and –Δ*E*/e voltage. The height of the conductance steps is proportional to the transition matrix element 
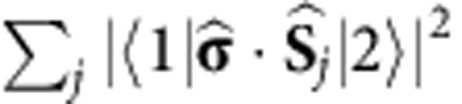
, where 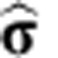 is the spin vector operator of the tunnelling electron and 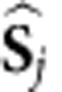 are the spin vector operators of the Fe atoms in the Fe_4_ molecule. The distribution of scattering strength between the four Fe atoms influences the relative height of the spin excitations steps. For simplicity, we assumed that scattering occurs with equal probability at any of the side atoms. Other ratios are possible but do not influence the voltage position of the conductance steps.

The calculated conductance spectra were used to fit experimental data by iterative computation of the spectrum while changing the exchange (*J*) and anisotropy (*D*) parameters in the spin Hamiltonian describing the Fe_4_ molecule (equation 1). Broadening of the conductance steps by finite temperature and bias modulation was taken into account. This led to step width of 400–600 μV. A linear slope was added to the conductance spectra following the fitting. Calculations that account for the out-of-plane magnetic field used a 9 T magnetic field applied at 33° from the molecular easy axis.

### DFT calculations

The relaxed structure of the Fe_4_ molecule adsorbed on the Cu_2_N surface was computed within the DFT framework with the Cp2k program package^28^,^29^ using the Dudarev simplified version^30^ of the DFT+U approach^31^,^32^ together with the PBEsol functional^33^.

The calculations followed methodology developed for SMMs on surfaces^34^ and used successfully for Fe_4_ molecules on Au surfaces^19^. The dispersion correction term (D3) was added to the energy^35^. The norm-conserving Goedecker-Teter-Hutter (GTH) pseudopotentials^36^ were used with GTH double-ζ polarized molecularly optimized basis sets for all atomic species^37^. The energy cutoff applied to the plane-wave basis sets was set to 500 Ry, in agreement with other studies on Cu_2_N^38^. The values of the parameter *U*, 4.1 eV on the Fe 3*d* and 3.0 eV on the O 2*p* orbitals were chosen following other calculations involving Fe_4_ molecules^39^. The convergence criterion for the self-consistent field method (SCF) energy was 1 × 10^−6^ Hartree. A threshold of 1 × 10^−3^ Hartree Bohr^−1^ for the atomic forces is considered sufficient for a reliable optimization. Optimization runs used a 0.17 eV smearing of the occupational numbers around the Fermi level to account for the metallicity of the surface, and to ease the convergence. For calculation of magnetic parameters, using the optimized geometry as a starting point, a *U* parameter may be added to the 3*d* states of the Cu atoms to ascertain the impact of the substrate on magnetic interactions within the Fe_4_ cluster.

The cluster and surface were relaxed separately before optimizing the adsorbed structure. The Cu_2_N surface slab consisted of 8 × 8 × 2 copper unit cells (width, breadth and thickness, respectively) and a layer of nitrogen atoms, positioned as starting geometry at 0.3 Å above the topmost copper layer in a c(2 × 2) manner. Out of the total four atomic copper layers, the bottom two were kept fixed to the Cu bulk positions during the geometry optimization. The experimental copper lattice constant of 3.615 Å was used^40^. The cluster was positioned on the relaxed Cu_2_N surface, leaning on two tert-butyl groups and the phenyl ring^19^. It was separated from its periodic images laterally by 13 and 14 Å, in *x* and *y* respectively, while the vacuum region extended 38 Å above the surface.

The exchange coupling constants in Fe_4_ were computed through the broken symmetry approach^25^,^41^,^42^ incorporating exchange interactions between nearest and next-nearest neighbours. For comparison to the giant spin Hamiltonian used in the main text, an equivalent nearest neighbour exchange coupling was calculated from the computed values of both the nearest and next-nearest neighbour coupling constants (See Supplementary Note 3 for details). The magnetic properties were evaluated on the optimized geometry with a tighter SCF convergence criterion of 5 × 10^−7^ Hartree, computing the energies of the determinants |uuuu〉, |duuu〉, |uduu〉 ,|uudu〉 and |uuud〉 (u stands for spin up (*m*=5/2), d for spin down (*m*=−5/2) on the four Fe ions). The interacting topmost layers of the substrate slab can be approximated as Cu_2_N, consequently, *U*_Cu_=5 eV^43^,^44^ was applied to all Cu atoms as reported previously in the literature. To verify the reliability of the computational protocol, the exchange coupling constants were also computed for the geometry determined from X-ray measurements and compared with experimental measurements (see Supplementary Table 1). To decouple the geometrical effects of adsorption from the electronic contribution of the surface, the magnetic couplings were computed for the relaxed molecule in the presence of the Cu_2_N surface and with the slab removed. In the distorted molecule compression was incorporated as a rigid shift of the upper tripodal ligand, with no further geometric relaxation.

## Additional information

**How to cite this article**: Burgess, J. A. J. *et al*. Magnetic fingerprint of individual Fe_4_ molecular magnets under compression by a scanning tunnelling microscope. *Nat. Commun.* 6:8216 doi: 10.1038/ncomms9216 (2015).

## Supplementary Material

Supplementary Information Supplementary Figures 1-5, Supplementary Tables 1-2, Supplementary Notes 1-5 and Supplementary References.
