# Supplementary material for: Magnetic fingerprint of individual Fe4 molecular magnets under compression by a scanning tunnelling microscope
Source: Nat Commun. 2015 Sep 11;6:8216. doi: 10.1038/ncomms9216 (PMC4579601; doi:10.1038/ncomms9216)
Supplement: Supplementary Information — Supplementary Figures 1-5, Supplementary Tables 1-2, Supplementary Notes 1-5 and Supplementary References. [file ncomms9216-s1.pdf]

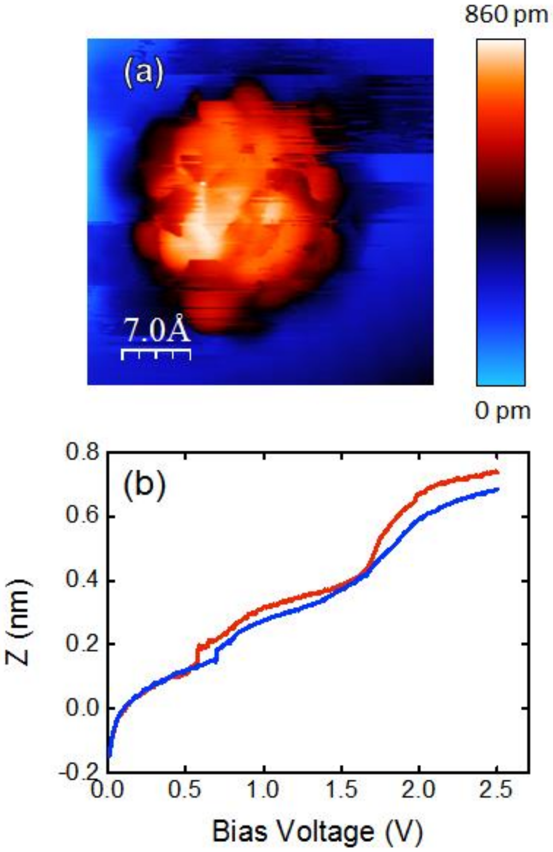

Supplementary Figure 1.| **Tip interactions with molecules on the Cu_2N surface.** **a**, A slow scan over a molecule with low tunnel current set point (3 pA), and high bias voltage (2 V), shows characteristic scars indicating transient tip-molecule interactions. Additionally, the image has a patchy appearance, indicating regions with qualitatively different tip-molecule interaction. These observations indicate that the tip is just barely in contact with the molecule for a sample bias of 2 V. **b**, Tip movement during transition from topographic to spectroscopic measurement. With the tip over a molecule, the bias voltage is ramped from +2.5 V to 10 mV with the STM feedback loop engaged and maintaining a current set-point of 3 pA. The tunnel gap reduces by almost 800 pm during the measurement. Given an estimated 750 pm tunnel gap over bare copper nitride and an apparent height of the molecules of 700-800 pm (both at a bias voltage of 2 V), the molecule must be compressed in the STM junction during a spectroscopic measurement. The hysteretic behaviour of approach curve (red line) and retraction curve (blue line) of the tip provides supporting evidence of contact events where ligands snap to the tip during compression, changing the conductance of the junction.

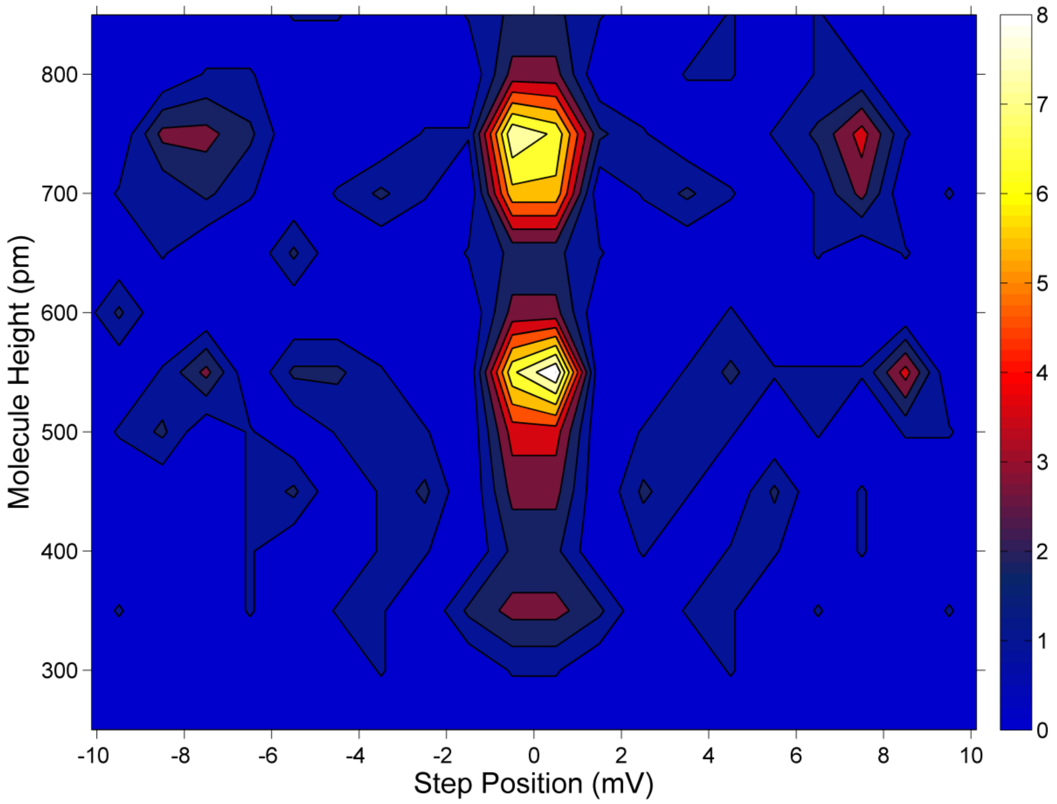

Supplementary Figure 2. | Excitation step positions extracted from molecules measured in the experiment. The observed steps are plotted in a 2D histogram binned by voltage position of the step and topographic height of the respective molecule. This figure matches Figure 3a in the main text (bins 0.75 mV and 50 pm, field 0 T), however, this plot shows raw data without symmetrization applied. The overall symmetry of the excitation pattern is preserved. Since energetic thresholds for spin excitations are symmetric with respect to the direction of current flow, this affords the opportunity to better fit peaks by having multiple measurements of the same excitation. Therefore, Figure 3a in the main text shows the symmetrized histogram where excitations are binned only by the absolute value of the voltage position. It is important, however, that the symmetrization process be checked to ensure a few coincident spurious steps picked up because of noise do not create an apparently robust looking peak which is not actually related to a spin excitation. This figure shows the raw data with excitations at $V < 0$ and $V > 0$ binned separately. During data acquisition, it is relatively easy to identify and reject coarse jumps resulting from noise or junction instability in the data. Consequently, few spurious steps are included in our results.

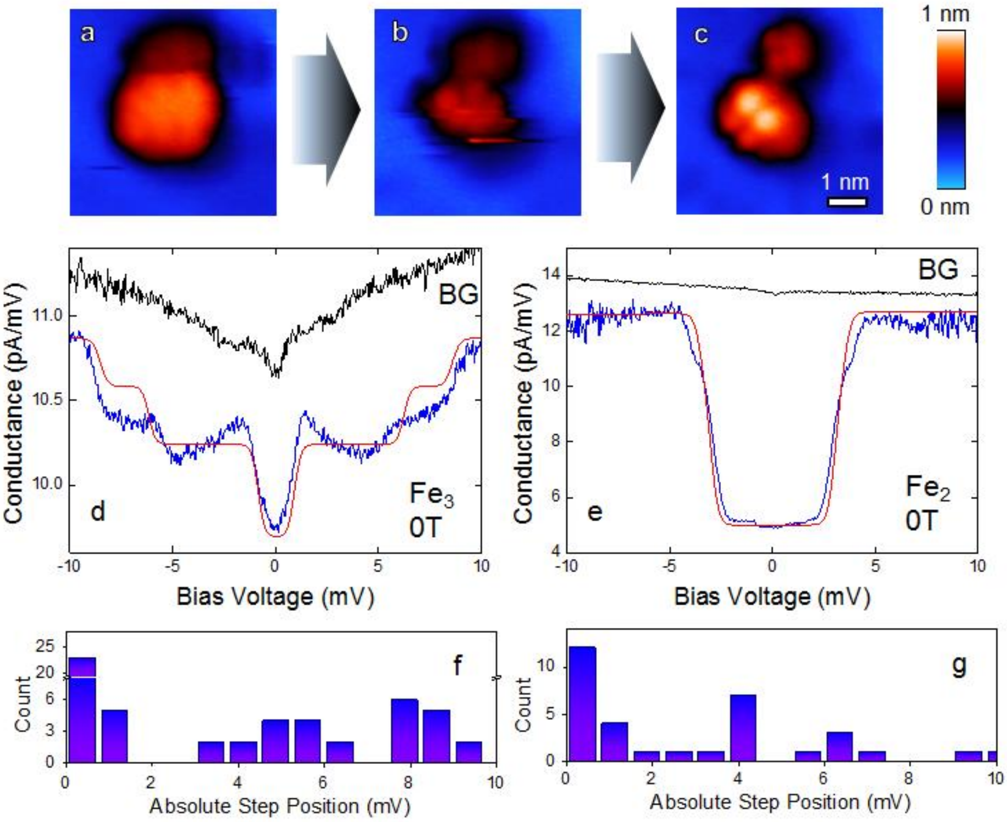

Supplementary Figure 3. | Observation of fragmentation and fragment spectra on the surface. a-c Constant-current topographs captured on the same molecular object at different times. The object is unstable; it changes during scanning and spectroscopy. **d, e,** Conductance spectra (blue lines) acquired after recording the topographs in a and c, respectively. Background spectra are shown in black. Red lines show calculated spectra using the effective spin Hamiltonian (GSH model) as described in the Supplementary Information Section IV. Fit parameters for d: three Fe ions, $D = -0.11$ meV (-0.90 cm^{-1}), $J = 2.3$ meV (18.4 cm^{-1}). Fit parameters for e: two Fe ions, $D = -43$ μeV (-0.35 cm^{-1}), $J = 3.1$ meV (25.0 cm^{-1}). **f,** Histogram showing the absolute value of spin excitation positions for molecules with topographic heights between 500 and 600 pm. The characteristic excitation energies match well with step positions calculated for magnetic fragments with three Fe ions, similar to d. **g,** Histogram similar to f but showing excitations for molecules with height below 400 pm. The peak positions match well with the peak positions in spectra computed for fragments with two Fe ions.

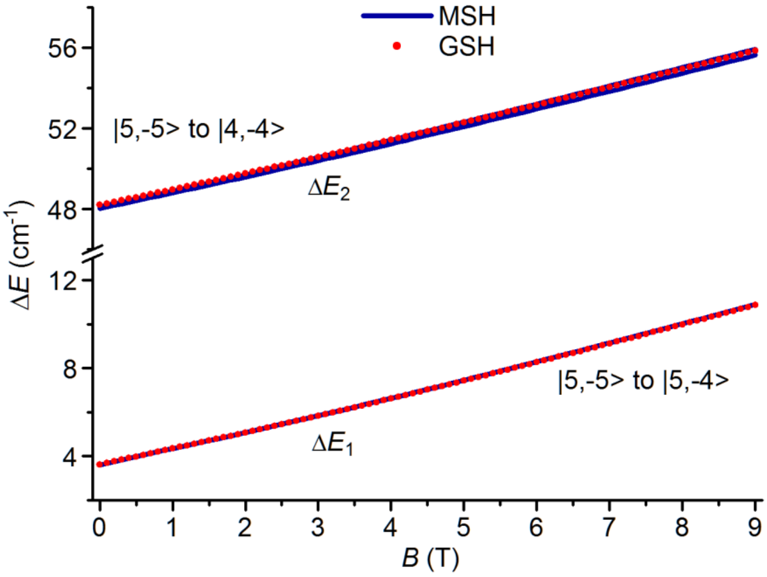

Supplementary Figure 4. | **Comparison of Giant and Multi Spin Hamiltonians.** Spin excitation energies predicted by the Giant Spin Hamiltonian (GSH) model used in the main text compared with those resulting from the more complete Multi Spin Hamiltonian (MSH) model reported in Supplementary Reference 1. The spin excitations between the ground state $|5,-5\rangle$ and the excited states $|5,-4\rangle$ and $|4,-4\rangle$ are shown for a magnetic field applied at an angle of 35 degrees from the easy axis of the molecule. $|4,-4\rangle$ labels two quasi-degenerate states. The MSH calculations are shown as blue lines and the GSH calculations as red dots. For details on parameters see Supplementary Note 3.

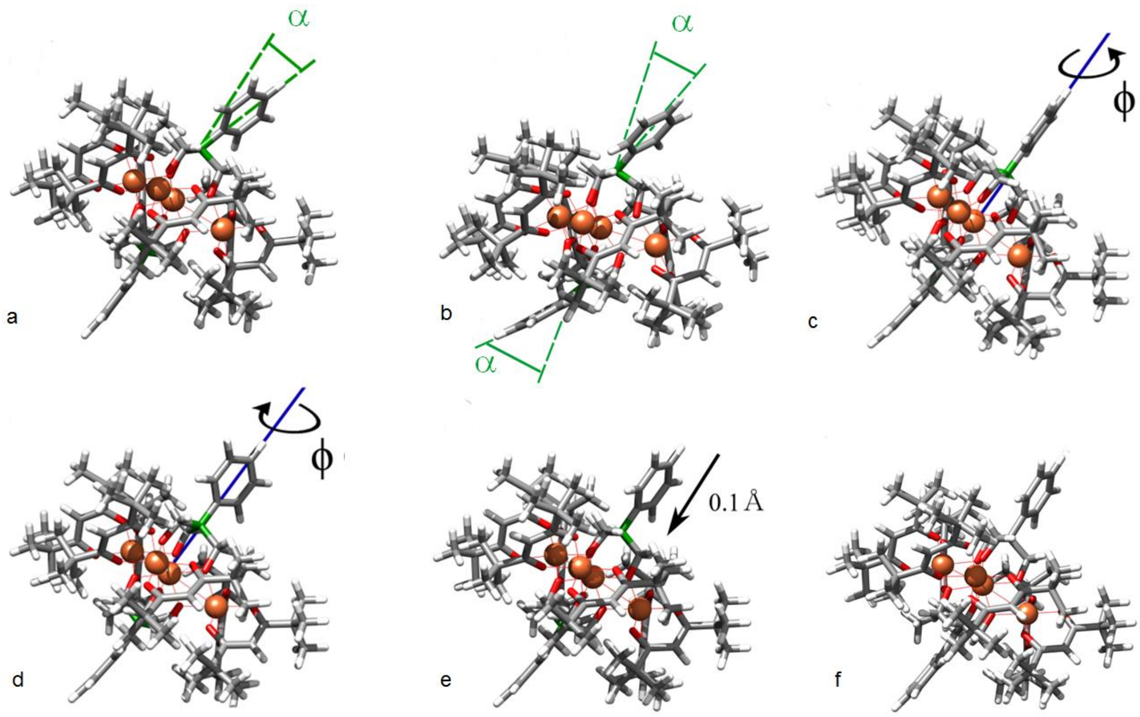

Supplementary Figure 5. | **Side view of the modeled distortions of Fe_4 molecule.** **a,** Distortion *A* is a tilt of one phenyl endgroup by 20°.The tilting angle, α, is measured as the angle between the para-H atoms and the quaternary carbon atom (green) in the tripodal ligand. **b,** Distortion *B* is a tilt of both phenyl endgroups by $\alpha = 40°$ applied to both rings. **c,** Distortion *C* is a rotation of the tripodal ligand far from the surface counter-clockwise by 20°. The axis of rotation is given as the axis passing through the central Fe ion and the quaternary carbon atom (green). **d,** Distortion D is the same type of rotation distortion as in c but with a clockwise rotation by 17°. **e,** Distortion E is a compression of the molecular core by rigid movement of the top tripodal ligand by 0.1 Å. The direction of movement is perpendicular to the plane of the four Fe atoms. **f,** Undistorted molecule. Atoms are Fe (orange), O (red), C (grey), H (white).

Fe_4	U_{Fe}, eV	U_O, eV	U_{Cu}, eV	J', meV (cm^{-1})	J'', meV (cm^{-1})
X-ray	4.1	3.0	-	2.08 (16.8)	0.06 (0.5)
Optimized	4.1	3.0	-	1.91 (15.4)	0.06 (0.5)
Extrapolated	4.1	3.0	-	1.69 (13.6)	0.06 (0.5)
Experimental				2.03±0.01 (16.37±0.12)	0.036±0.14 (0.29±0.11)
@Cu₂N	4.1	3.0	-	0.68 (5.5)	0.41 (3.3)
	4.1	3.0	5.0	1.09 (8.8)	-0.02 (-0.2)

Supplementary Table 1. | Theoretical results for the magnetic properties of the Fe_4 cluster in bulk and adsorbed. Magnetic couplings are computed using the broken symmetry approach [2] employing the exchange energy portion of the Multi Spin Hamiltonian (MSH) model described in reference 1 and Supplementary Note 4. Theoretical values for the exchange coupling in the molecule isolated from a surface are displayed for different molecular structures: the structure found in a bulk crystal (X-ray), the optimized structure found for a molecule isolated from the surface (Optimized) and the optimized structure for the molecule as adsorbed onto Cu_2N (Extrapolated). The experimental values for bulk Fe_4 crystals obtained in reference 1 are shown for comparison (Experimental). Excellent agreement is found between the X-ray computation and the experimental value. Two additional calculations (@Cu_2N) estimate the electronic impact of the surface one using no U parameter for the Cu atoms in the substrate, the other using U_{Cu} =5.0 eV. Both calculations use the structure of the molecule as optimized on the Cu_2N surface. All calculations use the same U values for Fe and O atoms. The positive values of J' and J'' determine antiferromagnetic and negative values ferromagnetic interaction. The values of J' and J'' presented are the mean values calculated from the individual couplings between each side atom and the central atom for J', and between each side atom pair for J''.

Fe_4		U_{Fe}, eV	U_O, eV	U_{Cu}, eV	J', cm^{-1}	J'', cm^{-1}
Impact of Structural Deformation (Extrapolated)	*No Distortion*	4.1	3.0	-	13.6	0.5
	Distortion A	4.1	3.0	-	13.6	0.5
	Distortion B	4.1	3.0	-	14.1	0.5
	Distortion C	4.1	3.0	-	26.6	0.6
	Distortion D	4.1	3.0	-	4.9	0.2
	Distortion E	4.1	3.0	-	24.9	0.7
Impact of Electronic Coupling to the Surface (@Cu₂N)	*No Distortion*	4.1	3.0	5.0	8.8	-0.2
	Distortion A	4.1	3.0	5.0	9.1	-0.1
	Distortion B	4.1	3.0	5.0	15.5	1.7
	Distortion C	4.1	3.0	5.0	23.0	0.0
	Distortion D	4.1	3.0	5.0	-1.7	1.8
	Distortion E	4.1	3.0	5.0	16.1	2.4

Supplementary Table 2. | Magnetic coupling in distorted molecules. Magnetic exchange coupling constants computed for each distorted configuration of the molecule shown in Supplementary Figure 5 and described in Supplementary Note 6. The different distortions are added to the molecular structure as optimized on Cu_2N with no further relaxation. Two sets of calculations are shown: one with no surface coupling (Extrapolated) and one including the electronic influence of the surface (@Cu_2N). The values of J' and J'' presented are the mean values calculated from the individual couplings between each side atom and the central atom for J', and between each side atom pair for J''.

Supplementary Note 1: Tip interaction with molecules

During scanning, even at very conservative tunnel junction set-points (3 pA, 2 V), the STM tip transiently interacts with the molecules on the surface. Commonly, transient noise spikes occur during typical scanning of the surface, sometimes shifting molecules or leading to fragmentation. In addition, slow scanning over an intact molecule produces a topographic image reminiscent of a molecular drag pattern on a surface (Supplementary Figure 1a). We interpret these features as sections of the molecule, likely protruding ligands, latching to the tip. As the tip scans, the latch breaks, and a new section of the molecule may latch on instead. This process can lead to a patchy image of the molecule with sharp jumps between sections where different ligands snap on to the tip.

The estimated tunnel gap over Cu_2N/Cu(100) at 2 V sample bias and 3 pA tunnel current is 750±50 pm. It was estimated by moving the tip into point contact with the surface at low voltage and monitoring the tip movement between the low-voltage tunnelling set-point and the set-point chosen here. Results from scanning indicate that the tip intermittently interacts physically with the top of the molecule. Given the typical height of the molecules measured relative to the surface in the constant-current mode of the STM (700-800 pm) this implies typical molecules are 1400-1600 pm tall on the surface. This height estimate matches well with the height to the top of the upper phenyl ring (1.7 nm) predicted by density function theory (DFT) calculations of Fe_4 on Cu_2N.

In spectroscopy, significantly reduced set-point voltage (10 mV vs 2V) necessitates a significant shift of the tip towards the sample surface to maintain the current set point. This is best illustrated by measuring the *z* piezo position as a function of voltage while keeping the STM feedback loop on (Supplementary Figure 1b). As the bias is reduced, the tip shifts towards the surface. Occasionally, jumps are noted, including hysteretic jumps in a complete bias sweep down and back up. This is another sign that ligands of the molecule attach physically to the tip. Between 2 V and 10 mV, the tip plunges 800 pm towards the surface, meaning that the tip is physically contacting and compressing the molecule.

Such strong tip movement indicates that this large, 3D molecule has an insulating character. Remarkably, despite the invasiveness of spectroscopy, many molecules survive several sets of measurements, permitting measurement of spin excitations.

Supplementary Note 2: Spectra of Magnetic Fragments

For large polynuclear complexes, fragmentation can occur in various ways and produce a variety of objects on the surface. Recent work[3] has provided insight into fragmentation for Fe_4 deposited on metallic surfaces. In the present work, we estimate that at minimum 30% of the molecules arrive intact on the surface following thermal deposition. Moreover during both scanning and spectroscopy, physical interactions of the tip with molecules occasionally lead to fragmentations, (Supplementary Figures 3a-c). Irrespective of what proportion of the molecules are broken by deposition or by the STM tip, fragmentation creates a challenge in the identification of spin excitation spectra.

For this reason a procedure is introduced in the main text that correlates observed spin excitations of a molecule with its height. This allows identification of the characteristic spin excitation spectrum of Fe_4 in the STM junction. Furthermore it allows some insight into the nature of typical fragments, which will be discussed here.

The 2D histogram in Figure 3a (main text) shows distinct patterns emerging for shorter molecules, implying the existence of common spectra associated with particular sized objects. Indeed, these peaks primarily stem from two spin excitation spectra that are markedly different from those of intact molecules (Supplementary Figures 3 d-e). We obtain good qualitative matches for these spectra by using the spin Hamiltonian introduced in the main text to describe intact Fe_4, but considering a reduced number of Fe ions in the molecule. Specifically, we consider clusters where one or two of the edge ions are removed and where uniaxial anisotropy is applied individually to each ion. This pattern of fragmentation is supported by observations of fragments created in secondary ion mass spectrometry (ToF-SIMS) experiments performed on films of Fe_4 molecules[4]. These measurements indicate that the most frequent fragments created during the SIMS measurement correspond to clusters with two or three Fe ions remaining. The fits to the experimentally observed spin excitations yield anisotropy energies varying between 43 and 190 μeV and exchange coupling strengths comparable to that found for the intact molecule (2.07 – 3.02 meV). The variation in excitation energies for qualitatively similar spectra support the conclusion that the other objects on the surface are also subject to tip-induced distortions that affect magnetic properties.

In Supplementary Figure 3 we present a sequence of images acquired from a single molecular object on the surface along with the associated evolution of the spin excitation spectrum. As the object undergoes topographic changes, the spectrum also changes. Initially the cluster exhibits a spectrum that matches well with the prediction for an Fe_3 cluster. After several scans and spectroscopic measurements the spectrum converts to one that is well described as an Fe_2 cluster.

Unambiguous interpretation of these spectra as belonging to fragments remains challenging. The exact configuration of the fragments remains unknown and is not readily extracted from highly variable, as well as generally non-distinct, topography. Additionally, while a naïve treatment of the magnetic model of the fragments (using the Fe_4 molecule simply with edge ions plucked off) provides a compelling fit to the observed spectra, it is accompanied by the overly strong

assumption that large changes in the magnetic coupling do not accompany large changes in observed topographical configuration.

Supplementary Note 3: Effective Spin Hamiltonian Model

The spin Hamiltonian introduced in the main text is a simplification of a more detailed multi-spin Hamiltonian (denoted MSH in the following) which was reported previously for Fe_4 molecules[1].

The MSH assumes threefold symmetry of the molecule and is given by

$$\hat{H}_{\mathrm{MSH}} = J'\hat{\boldsymbol{S}}_c \cdot (\hat{\mathbf{S}}_1 + \hat{\boldsymbol{S}}_2 + \hat{\mathbf{S}}_3) + J''(\hat{\mathbf{S}}_1 \cdot \hat{\mathbf{S}}_2 + \hat{\mathbf{S}}_2 \cdot \hat{\mathbf{S}}_3 + \hat{\mathbf{S}}_1 \cdot \hat{\mathbf{S}}_3) + g\mu_B \hat{\mathbf{B}} \cdot \hat{\mathbf{S}}_{\mathbf{T}} + \sum_{i=1,2,3,c} \hat{\mathbf{S}}_i \cdot \mathbf{D}_i \cdot \hat{\mathbf{S}}_i + \sum_{i \neq j} \hat{\mathbf{S}}_i \cdot \mathbf{D}_{ij} \cdot \hat{\mathbf{S}}_j \quad (1)$$

where $\hat{\mathbf{S}}_i$ are the spin vector operators of the three side Fe ions (i = 1,2,3) and $\hat{\mathbf{S}}_c$ is that of the central ion. The first term describes Heisenberg exchange interaction between the side ions and the center ion with strength J'. The second term describes next-nearest neighbour exchange between the side ions with strength J''. The third term describes Zeeman energy introduced by the external magnetic field, $\mathbf{B}$; $\hat{\mathbf{S}}_{\mathbf{T}}$ is the vector operator of the total spin, g is the Landé g-factor and μ_B is the Bohr magneton. The fourth term describes the quadratic magnetic anisotropy of all Fe ions; the $\mathbf{D}_i$'s are second-rank tensors that account for second-order magnetic anisotropy of Fe ions in both magnitude and orientation. The fifth term describes anisotropic spin-spin interactions between the ions using a second-rank tensor $\mathbf{D}_{ij}$. It accounts, for example, for dipolar interactions.

The simplified Hamiltonian used in the text is a giant-spin Hamiltonian, denoted GSH in the following, and is given by

$$\hat{H}_{\mathrm{GSH}} = \sum_{i=1,2,3} J\hat{\boldsymbol{S}}_{\mathrm{i}} \cdot \hat{\boldsymbol{S}}_c + g\mu_B \hat{\mathbf{B}} \cdot \hat{\mathbf{S}}_{\mathrm{T}} + D\hat{S}_{\mathrm{T},z}^2 \quad (2)$$

It keeps the nearest-neighbour interaction and Zeeman energy terms. The magnetic anisotropy is reduced to account only for its leading term: easy-axis second-order anisotropy parallel to the axis of the tripodal ligands. Here, the anisotropy is evaluated for the z-component of the total spin of the molecule, $S_{\mathrm{T},z}$, and it has been assumed that the same anisotropy constant applies for both the $S_T = 5$ and $S_T = 4$ multiplets. The following comparison to the MSH model shows that this simplification is possible. Next-nearest neighbour exchange and dipolar interactions were not included as their energy contributions to the spin states are small. Notice that this will in general cause J in the GSH model to differ from J' in the MSH model. This has no practical consequences,

since the experimentally observable quantity is the energy gap between the $S_T = 5$ and $S_T = 4$ multiplets, which amounts to $\Delta = \frac{5}{2}J$ in the GSH and to $\Delta = \frac{5}{2}J' - \frac{15}{2}J''$ according to the MSH. We take this difference into account when comparing the exchange energies obtained here and those reported elsewhere. We find that the simplifications made in the GSH introduce only minute deviations of the eigenenergies for the spin states of the lowest $S_T = 5$ and $S_T = 4$ multiplets compared to the MSH model, Supplementary Figure 2. The deviations do not exceed 50 μeV up to 9 T magnetic field and are therefore smaller than the accuracy of the inelastic tunnelling spectroscopy which is 100 μeV.

To demonstrate this, the GSH model is benchmarked against the MSH using a parameter set taken from a previous publication[1]. In accordance with experimental magnetic and structural studies on the highest-symmetry (D_3) derivative of this series, nearest-neighbour and next-nearest neighbour Heisenberg interactions were set to J' = 2.05 meV (16.51 cm^{-1}) and J''= –77 μeV (–0.62 cm^{-1}), yielding Δ = 5.69 meV (45.93 cm^{-1}). Point-dipolar couplings were calculated from the observed Fe-Fe separations. Single-ion tensors $\mathbf{D}_i$ were introduced at appropriate Euler angles in the molecular reference frame, which has *z* normal to the metal plane and *y* directed along the line joining the central ion to a side ion. The single-ion tensor for the central Fe ion was assumed to be axial along *z* with $\mathbf{D}_c$ = –76 μeV (–0.61 cm^{-1}); single-ion tensors for the side Fe ions were assumed to be rhombic with $\mathbf{D}_{1,2,3}$ = 95 μeV (0.77 cm^{-1}) and a transverse anisotropy parameter $\mathbf{E}_{1,2,3}$ = 11 μeV (0.09 cm^{-1}); their hard axis was set normal to *z* and their intermediate axis directed toward the central ion. This model yields a ground spin multiplet that closely approximates a S_T = 5 state. The next higher spin states are two quasi-degenerate multiplets that closely resemble S_T = 4. Finally, an angle θ = 35° between the applied magnetic field and the *z* axis was considered in the calculations while the *g* factor was assumed to be isotropic and equal to 2. For $\theta \neq 0$ the spin levels depend on the orientation of the in-plane component of the magnetic field (i.e. the polar angle ϕ); these energy differences are however of the order of 1 μeV (10^{-5} cm^{-1}) and can be neglected here. We note that, although the MSH contains terms that do not commute with $\hat{\mathbf{S}}_T^2$ and $\hat{\mathbf{S}}_{T,z}$, the dominant Heisenberg exchange and the small θ value make S_T and $m = <\hat{\mathbf{S}}_{T,z}>/\hbar$ good quantum numbers. Thus, for simplicity the spin states are labeled as $|S_T, m>$.

Supplementary Figure 4 plots the energies for the two lowest-energy spin excitations that can be observed in conductance spectra. The excitations are between the ground state $|5,-5\rangle$ and the excited states $|5,-4\rangle$ and $|4,-4\rangle$. The calculated spin excitation energies increase nearly linearly with increasing magnetic field, and feature a slope that is lower than that expected for simple Zeeman splitting due to the presence of the magnetic anisotropy.

The parameters of the MSH model can now be mapped to the parameters used in the GSH model. The zero-field transition energy between $|5,-5\rangle$ and $|5,-4\rangle$ amounts to $9|D(S_T = 5)|$ in the GSH model and requires $D(S_T = 5)$ = –50 μeV (–0.402 cm^{-1}). The zero-field energies resulting from MSH further indicate that the simplification to use only one parameter, D, in the GSH model is valid. A remarkable agreement between MSH and GSH is in fact achieved by setting $D(S_T = 4) = D(S_T = 5)$ and using *J* = 2.28 meV (18.37 cm^{-1}), so that Δ = 5.69 meV (45.93 cm^{-1}). The red dots in Supplementary Figure 4 show the field evolution of spin excitation energies produced by the GSH model. It is evident that the MSH and GSH model agree very well with each other in the range of parameters and magnetic fields used here. Alternative parameter sets in the MSH, such as those reported in Ref. 5, yield similar results.

In conclusion, the GSH provides a very simple and effective model that accurately describes the two spin excitations observable in inelastic tunnelling spectra. It also facilitates quantitative analysis of the observed spin excitations with only two adjustable parameters: D and J.

Supplementary Note 4: Prevalence of Excitations to Other Multiplets

The possibility that other excitations, besides the excitation to the S_{T} = 4 multiplet, cause the 7.5 mV step was considered. The most probable alternative, and next highest energy excitation is to the lowest lying state in a higher spin multiplet with S_{T} =6. The presence of this excitation in the spectra shown in the main text may be ruled out based on the magnetic field dependent shift of the spin excitations that indicates that the excitation steps shift to larger energy with increasing field.

However, due to the challenge of taking data on a molecule prone to fragmentation under STM measurement only a subset of the molecules used in the correlation analysis of Figure 3 could be measured at different magnetic fields. Consequently, it is possible that a sub population of molecules investigated in Figure 3 exhibit the transition to the S_{T} = 6 multiplet. Here we investigate this possibility to place an upper bound on the proportion of molecules that may fall into this category.

The excitations to S_T = 6 or 4 are associated with inelastic excitation on the central or on an edge atom respectively. The compactness of the Fe_4 molecule did not allow topographic identification of the individual Fe ions within the molecules. Addressing only one atom is therefore unlikely for any one spectrum. Consequently, we expect spectra to show spin excitations of all Fe ions and both excitations to be visible.

Assuming that the spin excitation observed at 7.5 meV in the intact molecules was caused by an excitation to S_{T} = 6, this would place the excitation to S_{T} = 4 at approximately 4 meV. In this scenario the exchange coupling strength between the central and the side Fe ions would be slightly reduced compared to bulk samples of Fe_4. Consequently, we would expect the spectra and histogram to show three steps, specifically with a gap $\Delta_{4,6} \approx 0.3\,J - 0.12$ meV, between the second and third steps. Only one spectrum was obtained approximately matching this description on molecules that remained intact and featured a height commensurate with intact molecules. Therefore, we conclude that the majority of Fe_4 molecules placed in the STM junction show only the excitation to S_T = 4 in spectra recorded between ±10 mV and consequently feature a boosted exchange interaction.

Supplementary Note 5: Computation of Exchange in Distorted Molecules

Several geometrical rigid distortions have been tested to simulate the structural modification of the Fe_4 molecule in the STM junction. The distortions are: tilting of the phenyl endgroups on the tripodal ligands, compression of the molecular core by movement of the tripodal ligands and compression-induced rotation of these ligands.

In detail, we consider the folding of the phenyl rings of the tripodal ligands towards the cluster core in two ways. In distortion A, only the phenyl ring far from the surface is shifted with a tilting angle, α, of 20° (Supplementary Figure 5a), while in distortion B both phenyl rings are tilted by α = 40° (Supplementary Figure 5b). We also consider an alternative effect of compression on the Fe-O-Fe bond angles that occurs by tilting of the planes defined by the bonds. Under such compression, the planes of the bonds should tilt away from the axis of the molecule. We mimic this in distortion C by considering a clock-wise rotation of the upper tripodal ligand around the molecular axis by an angle $\phi = 17°$ (Supplementary Figure 5 c) with respect to the undistorted structure. For completeness, we consider a distortion, D, featuring a counter clock-wise rotation of ϕ = 20° (Supplementary Figure 5d). In distortion E we consider a planar compression of the molecular core by moving the top tripodal ligand towards the plane of the four Fe ions by 0.1 Å.

The distortions are applied on the Fe_4 cluster already optimized on Cu_2N, without any further relaxation of the geometry. The magnetic exchange coupling constants for each distorted molecule have been computed in the extrapolated geometry, with the molecule isolated from the surface. This provides a clearer estimate of the effects of the geometrical distortions without making assumptions about how the molecule is compressed onto the surface.

The results for the undistorted molecule, distortions A to D, and the simple compression distortion introduced in the main text (here labeled E) are compiled in Supplementary Table 2. We find that, in addition to the simple compression, the only distortion that causes a large increase in exchange coupling is a very large counter clock-wise rotation. However, distortions C and D are extreme, corresponding to ≈100 pm shifts of each oxygen attached to the base of the top tripodal ligand. Furthermore, the counter-clockwise rotation direction provides a simulation of an extension force along the molecular axis, rather than a compressive force. The only distortion that has a geometrical effect commensurate with the experimental exchange boost is the small (10 pm) compression introduced in the main text. Indeed, the simple compression alone has sufficiently large impact on both J' and J'' for a realistic amplitude distortion to describe the experimental value of J.

Calculations incorporating both distortions and the copper slab offer the opportunity to check if the trend of boosted exchange is preserved when the electronic influence of the surface is included. These calculations push the state-of-the-art and are only possible implementing approximations which necessarily reduce the computational accuracy. The molecules are considered to rest on the surface exactly as the relaxed Fe_4 molecule with no additional relaxation for all the distortions. In the case of distortion *B*, the molecule has been manually tilted towards Cu_2N, so that the phenyl

ring lies on the surface and the angle between the molecular axis and the surface normal is reduced to 14.0°. The lack of relaxation reduces the accuracy of the description of the molecule-surface interaction, however an approximate estimation of the surface is possible. Results are included in the second half of Supplementary Table 2.

As in the case of the relaxed and non-distorted molecule, the surface is found to, in general, reduce exchange coupling. The same trend in J' is however observed for the deformations: distortions *B, C* and *E* retain the increased exchange compared to the undistorted molecule on the surface while only distortion *D* exhibits reduced exchange. The surface also has an effect on the next nearest neighbour coupling J''. However, the evaluation of these weaker interactions is subject to a proportionally larger uncertainty introduced by the approximate treatment, and so, no strong conclusion can be drawn about J''.

The calculations performed overall indicate that compressive distortions have the tendency to increase exchange coupling, while the electronic effects of the surface seem to suppress it. This supports the conclusion that the enhanced excitation energy observed in the experiment mostly likely results from the structural changes in the molecule induced by the tip.

Supplementary References

1. Accorsi, S., et al. Tuning anisotropy barriers in a family of tetrairon(III) single-molecule magnets with an S = 5 ground state. *J. Am. Chem. Soc.* **128**, 4742–4755 (2006).

2. Noodleman, L. and Norman, J. G. J. The Xα valence bond theory of weak electronic coupling. Application to the low-lying states of $Mo_2Cl_8^{4-}$. *J. Chem. Phys.* **70**, 4903–4906 (1979).

3. Malavolti, L., et al. Magnetic bistability in a submonolayer of sublimated Fe4 single-molecule magnets. *Nano Lett.* 15, 535–541 (2015).

4. Margheriti, L., et al. Thermal deposition of intact tetrairon(III) single-molecule magnets in high-vacuum conditions. *Small* **5**, 1460–1466 (2009).

5. Vergnani, L., et al. Magnetic bistability of isolated giant-spin centers in a diamagnetic crystalline matrix. *Chem. Eur. J* **18**, 3390–3398 (2012).
